# MACI - a new era?

**DOI:** 10.1186/1758-2555-3-10

**Published:** 2011-05-20

**Authors:** Matthias Jacobi, Vincent Villa, Robert A Magnussen, Philippe Neyret

**Affiliations:** 1 Hôpital de la Croix Rousse, Centre Albert Trillat, service orthopédie, pavillon R, Groupement Hospitalier Lyon Nord, 103, Grande Rue de la Croix Rousse, 69004 Lyon, France; 2 Orthopädie am Rosenberg, Rorschacherstrasse 150, 9000 St. Gallen, Switzerland

## Abstract

Full thickness articular cartilage defects have limited regenerative potential and are a significant source of pain and loss of knee function. Numerous treatment options exist, each with their own advantages and drawbacks. The goal of this review is to provide an overview of the problem of cartilage injury, a brief description of current treatment options and outcomes, and a discussion of the current principles and technique of Matrix-induced Autologous Chondrocyte Implantation (MACI). While early results of MACI have been promising, there is currently insufficient comparative and long-term outcome data to demonstrate superiority of this technique over other methods for cartilage repair.

## Introduction

Isolated chondral or osteochondral lesions of the knee are regularly found in a population undergoing knee arthroscopy[1,2]. Origins include traumatic injuries, abnormal joint loading, and osteochondritis dissecans among others. Cartilage lesions are often found in association with anterior cruciate ligament injuries, dislocations of the patella, limb malalignment, patellar maltracking and following significant meniscectomy[2-5]. Determining the ideal treatment of these lesions is problematic because it is often difficult to determine whether the patient's symptoms are caused by the cartilage lesion or by an associated pathology. It has been shown that even in isolation, these lesions may lead to significant pain and disability[6].

Damaged articular cartilage has limited or no healing capacity due its relative metabolic inactivity and lack of blood supply that permits only a limited response to injury[7,8]. These lesions may progress to generalized osteoarthritis over time[9]. Repairing isolated full-thickness cartilaginous defects has been therefore proposed to treat symptoms and prevent the development of osteoarthritis. Successful early treatment of these lesions would be of great benefit to patients as well as the health care system, as long-term morbidity and consequent high use of health service resources could be avoided[10]. Imaging studies facilitate the diagnosis of isolated cartilage lesions. MRI has been established as the diagnostic gold standard and should be considered when a chondral injury is suspected[11,12].

Many treatment options have been developed during the last decades to repair damaged articular cartilage[13]. The techniques can be grouped as bone marrow stimulation techniques such as drilling[14], abrasion[15], microfracture[16] and autologous matrix induced chondrogenesis (AMIC)[17]; direct chondral replacement techniques such as mosaicplasty[18], fresh osteochondral allograft transplantation[19], and periosteal transplantation[20]; and culture-based techniques such as Autologous Chondrocyte Implantation (ACI)[21] and Matrix-induced Autologous Chondrocyte Implantation (MACI)[22]. Each of these procedures can be performed in association with new techniques, materials, or growth factors, leading to the description of a huge number of treatment options that have been used in experimental and clinical settings[23].

This review will provide an overview on the historical development of cartilage repair. The main focus will be on the MACI technique and its variants and the clinical evidence for its use compared to other cartilage repair procedures.

### Historical development of cartilage repair

As early as 1743 William Hunter stated that "ulcerated cartilage is a troublesome thing, once destroyed it is not repaired"[24]. In 1853 James Paget reported that there are "no instances in which a lost portion of cartilage has been restored, or a wounded portion repaired with new and well formed cartilage"[25].

In 1941, Magnuson was among the first to describe operative treatment of diseased portions of articular surfaces. His concept of complete debridement of the knee joint for osteoarthritis was novel and original. He debrided the joint, including removal of osteophytes and a kind of abrasion was done[26]. Pridie took up the principle of Magnuson and described his own technique in 1951[14]. On previously eburnated joint surfaces he performed drill holes via an open approach, perforating the subchondral lamina. He observed the growth of repair tissue and pain relief in his patients. This technique has shown to provide significant symptomatic improvement in 75% of patients at a mean of eight years following surgery[27]. Later, Johnson popularized arthroscopic abrasion arthroplasty, which was also based on Magnuson's experiences[15]. The subchondral lamina was removed with an arthroscopic burr, releasing mesenchymal stem cells into the lesion and promoting the formation of repair tissue. Abrasion was often combined with lavage, debridement and partial meniscectomy. The technique was noted to relieve pain for up to 5 years, with better results noted in younger patients[28].

In the early 1990s, Steadman described the Microfracture technique[16,29]. He performed multiple perforations of the subchondral lamina with an arthroscopic awl. Possible advantages of this technique include avoidance of heat necrosis, which might be associated to the use of a drill burr, and preservation of enough subchondral bone to avoid any risk of collapse. Additionally, the development of angled awls allowed access to regions that were difficult to reach arthroscopically with a drill or burr. Encouraging results have noted at medium-term follow-up, especially in younger patients; however, around 20% of patients are generally not satisfied after five years[30-32].

A final variant of bone marrow stimulation is the autologous matrix induced chondrogenesis (AMIC) technique published in 2005 by Behrens[17]. Following microfracture, a collagen scaffold is placed over the defect, holding the blood clot and mesenchymal stem cells released from the marrow in place over the defect, theoretically aiding the cartilage repair process[17]. Their group has reported good early results at a mean of three years post-operative[33].

Regardless of the specific technique, bone marrow stimulating procedures generally induce the formation of fibrocartilage as repair tissue[30,34]. This tissue has limited mechanical resistance compared with hyaline cartilage, potentially leading to earlier degradation and subsequent failure[35]. For this reason, alternative procedures have been developed in an attempt to create hyaline repair tissue.

Replacement of the injured cartilage is one such approach to restore the joint surface. Osteochondral Autograft Transplantation (OAT) and mosaicplasty are techniques in which the injured cartilage is replaced with osteochondral plugs taken from non-weightbearing portion of the joint. Hangody initially described this technique in the 1990s and has demonstrated successful transplantation of autologous hyaline cartilage[18,29,36]. A single plug may be sufficient for small lesions, while larger lesions frequently require several grafts. Depending on lesion size and location, an open or arthroscopic approach may be undertaken. Alternatively, fresh osteochondral allografts can have been proposed for full-thickness osteochondral defects, particularly for defects greater than 3 cm in diameter or 1 cm in depth of the femoral condyles[19]. The success of mosaicplasty is limited in these large lesions due to donor site morbidity and healing seams at the recipient site. Results of mosaicplasty are often satisfactory in the medium term with slight deterioration over time[37,38].

Based on the work of Bentley and Greer, there has been increasing interest in the ability of transplanted chondrocytes to reform damaged articular cartilage[5]. Autologous chondrocyte implantation (ACI) was the first example of tissue engineering in cartilage repair. ACI was first utilized in humans in 1987 and first reported by Brittberg in 1994[21]. In this technique, cultured chondrocytes are injected under a periosteal cover, which is sutured onto the defect. In order to contain the cultured chondrocytes in the defect, a watertight suture of the periosteum to the surrounding cartilage is required. A preliminary surgery is necessary to harvest autologous cartilage, followed by several weeks of cell culture. The technique changed over time to the second generation ACI, in which the periosteal membrane was replaced by a collagen scaffold[39]. ACI has been reported to yield good outcomes in a large percentage of patients as long as 10 to 20 years after implantation[40].

A later development was to culture the autologous chondrocytes on a three-dimensional artificial scaffold. This third generation chondrocyte transplantation technique, commonly referred to as the matrix-induced autologous chondrocyte implantation (MACI) technique, is detailed in the next section[22,41].

During the last two decades tremendous effort has been undertaken to shorten cell culture, engender other cells with chondrocyte-like characteristics, and to produce tissue easier for the surgeon to implant[31,42-44]. However few of these experimental developments have reached clinical application.

## MACI procedure

### Principle

**Figure 1:**
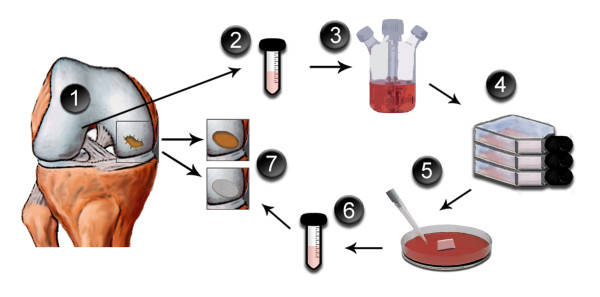
**The MACI procedure**. (1) Initial arthroscopy with evaluation of the injured cartilage and harvest of a full-thickness cartilage biopsy; (2) the biopsy is sent in a sterile and cooled container to the cell culture laboratory; (3) the cartilage is enzymatically digested; (4) expansion of the chondrocytes in monolayer culture for about four weeks; (5) the cells are seeded onto the scaffold a few days before implantation; (6) the engineered implant is sent back to the surgeon in a sterile container; (7) definitive surgery with debridement of the injured cartilage followed by implantation of the MACI-implant, which is trimmed to fit the defect size and glued with a thin layer of fibrin glue.

The technical difficulty and need for a relatively large arthrotomy associated with the traditional ACI procedure as well as a desire to improve the subsequent repair tissue have spurred the desire to develop an easier, more effective method of implanting cultured chondrocytes into the knee joint[41,45]. The principle is to culture autologous cells onto a three dimensional biocompatible scaffold, which is then implanted into the defect (Figure 1). As with the ACI technique, an initial arthroscopic harvest is necessary to obtain chondrocytes for culture. A full-thickness cartilage specimen is generally taken from a non-weightbearing region of the knee joint such as the area around the intercondylar notch or the lateral border of the trochlea. This initial surgery is also an opportunity to evaluate the lesion and to confirm the indication.

### Graft preparation

The cartilage biopsy is enzymatically digested to release the chondrocytes entrapped in the collagen matrix. Expansion of chondrocytes is generally performed in monolayer culture to obtain fifteen to twenty million cells over about four weeks. A few days before implantation, the expanded chondrocytes are seeded onto a biodegradable scaffold [41]. A common problem in monolayer expansion of chondrocytes is the dedifferentiation of cells. Seeding of the cells onto a three dimensional collagen I/III scaffold can lead to at least partial redifferentiation. It has been shown that cells grown on the scaffold can synthesize typical chondrocyte matrix components including glycosaminoglycans, chondroitin sulfate and type II collagen. The S-100 protein, which is a cytoplasmic marker of chondrocytes, has also been detected[22,46,47].

Several different scaffolds are currently used in clinical settings. The matrix-induced ACI (MACI) method (Genzyme Biosurgery, Cambridge, Massachusetts, USA) relies on a purified and cell-free porcine collagen scaffold [41]. Hyalograft-C (Fidia Advanced Biopolymers, Abano Terme, Italy) utilizes a scaffold based on hyaluronic acid[48]. Novocart 3D (TETEC Tissue Engineering Technologies AG, Reutlingen, Germany) uses a collagen-chondroitin-sulfate based membrane.[49] BioSeed-C (Biotissue Technologies, Freiburg, Germany) relies on a fibrin and polymer-based scaffold of polyglycolic/polylactic acid and polydioxanone[50]. Cartipatch (Tissue Bank of France, Lyon, France) utilizes an agarose-alginate hydrogel scaffold[51]. Only the MACI technique is currently available in the United States. It has the largest clinical experience and the majority of published reports, including two randomized clinical trials, refer to this technique. For these reasons, our review will focus on the MACI technique.

### Surgical technique

Implantation of the scaffold can be performed in an open or arthroscopic manner depending on the size and location of the lesion. The cartilage defect is first debrided down to the calcified cartilage layer without penetration of the subchondral lamina. The border of the lesion is then prepared to achieve stable and vertical edges. The cultured cartilage implant must then be trimmed to exactly match the defect size and not protrude beyond the margins. The implant is then fixed into the defect with a minimal amount of fibrin glue. The cell-seeded side is placed facing the subchondral bone. Pressure is applied for several minutes to ensure fixation. In uncontained and large defects the use of biodegradable bone anchors or limited suture fixation may be necessary to avoid graft delamination[41]. Surgical time is typically shorter than traditional ACI as implantation and fixation are facilitated. Therefore it may be easier to perform concurrently with other interventions such as ligamentous reconstruction, bone grafting or high tibial osteotomy[52].

### Rehabilitation

The goal of rehabilitation is to safely restore knee function including range of motion, muscle strength, and coordination while protecting the implanted graft during its maturation. After a short initial immobilization period, continuous passive motion (CPM) is recommended as it has been shown to stimulate synthesis of glycosaminoglycans, chondroitin sulfate and type II collagen[53]. Typically, eight to twelve weeks of limited weight bearing and progressive range of motion are advocated, followed by progressive advancement of activity level. A randomized controlled trial comparing standard (eleven weeks) versus accelerated (eight weeks) rehabilitation found no negative influence of accelerated rehabilitation at three months[54]; however, mid- and long-term results are not available. Full return to sports activities is generally not permitted until 18 months after surgery.

## Results and State of the Evidence

### MACI Procedure

MACI has been reported to be a successful method to treat symptomatic isolated cartilage defects. Many case series (level 4 evidence) are available reporting improvement of pain and function after this procedure in short- and medium-term follow-up[22,39,48,49,55-60]. Relevant clinical studies are summarized in table 1. MRI evaluation generally demonstrates filling of the cartilage defect; however, some hypertrophy, incomplete filling, and limited integration with surrounding normal cartilage has been noted at up to 60 months postoperatively[39]. No data is currently available demonstrating that this procedure prevents or delays the development of osteoarthritis. Unfortunately, the vast majority of clinical evidence regarding MACI is based on small case series using a variety of techniques on heterogenous patient populations, the results of which are evaluated with a plethora of incomparable outcome measures. These factors impair the ability to compare results between studies, which are often contradictory[61].

**Table 1:** Clinical Results of MACI

Author	Patients	Follow-up (months)	Study design	Major Findings
Basad [69]	60	24	Level I RCT versus Microfracture	Significantly larger improvements in Lysholm, Tegner and ICRS scores were noted in the MACI group

Visna[71]	50	12	Level I RCT versus microfracture	Significantly larger improvements in Lysholm, Tegner and IKDC scores were noted in the MACI group

Kon[70]	80	60	Level II Prospective cohort versus microfracture	Significantly larger improvement in IKDC score was noted in the MACI group. Results deteriorated from 2 to 5 years in microfracture but not MACI group

Bartlett [45]	47	12	Level I RCT versus ACI	Significant improvements in Cincinnati score and VAS were noted in both group - no significant differences between the two groups

Wondrasch[60]	31	24	Level 1 RCT Standard versus accelerated rehabilitation	Significant improvements in IKDC, KOOS, Lysholm and Tegner scores were noted in both group - no significant differences between the two groups

Behrens [22]	34	34	Level IV Case series	Significant improvements in Meyer, Lysholm and ICRS scores

Ebert[64]	35	120	Level IV Case series	Significant improvements in KOOS, SF-36 and MRI composite scores

D'Anchise[48]	35	24	Level IV Case series	Significant improvements in VAS, IKDC, Lysholm and Tegner scores

Postoperative complications and adverse events associated with the MACI procedure have been reported in clinical studies, including tissue hypertrophy, infections, the need for subsequent surgical procedures, and treatment failure[22,39,45,62,63]. Reported incidence rates of postoperative complications are generally low (0-6.3%)[39,45,63]. One of the more common problems is hypertrophy of the repair site, which can be arthroscopically debrided[45].

### Comparative Studies

In spite of significant research, none of the techniques described above consistently demonstrate superior clinical outcomes compared to the others[61,64], with some authors noting improved results with ACI[65] or mosaicplasty[66] compared with marrow stimulation techniques and other noting no difference[30,34,59]. One randomized controlled trial comparing mosaicplasty to ACI favored mosaicplasty[67] another favored ACI[68].

Four prospective comparative studies are currently available that compare MACI to another cartilage repair procedure. Basad et al [69], Kon et al[70], and Visna et al[71] compared various types of MACI to marrow stimulation techniques. Basad et al and Visna et al found the MACI procedure to be superior to marrow stimulation techniques at short-term follow-up (1 to 2 years)[70,71]. Kon et al noted some deterioration of microfracture results between 2 and 5 years post-operative, while MACI results were unchanged[69], Bartlett et al compared MACI with traditional ACI and noted no significant differences between the two groups[45]. Importantly, there are currently no studies comparing patients treated with MACI to an untreated control group.

## Conclusions

The MACI technique is a safe procedure for the treatment of symptomatic articular cartilage lesions. It is a two-step procedure relying on expensive cell culture techniques. Technically, it facilitates surgery and reduces operative time and the need for open surgery compared to traditional ACI. Symptomatic improvement has been shown at short- and medium-term follow-up. Available comparative studies suggest that MACI may be superior to marrow stimulation techniques, but long-term outcome data and comparisons against conservative management are lacking. No data are currently available demonstrating its capacity to prevent or delay the onset of osteoarthritis. The role of MACI in cartilage repair surgery remains a subject of intense investigation and has yet to be fully defined.

## List of abbreviations

ACI: Autologous chondrocyte implantation; MACI: Matrix-induced Autologous Chondrocyte Implantation; AMIC: Autologous Matrix Induced Chondrogenesis

## Competing interests

The authors declare that they have no competing interests.

## Authors' contributions

MJ & VV wrote the manuscript. MJ prepared the artwork. RAM & PN edited and proofread the manuscript. All authors have read and approved the final manuscript.
